# Transdermal Delivery by Iontophoresis

**DOI:** 10.4103/0250-474X.40324

**Published:** 2008

**Authors:** Swati Rawat, Sudha Vengurlekar, B. Rakesh, S. Jain, G. Srikarti

**Affiliations:** Smriti College of Pharmaceutical Education, Indore - 452 010, India

**Keywords:** Electro repulsion, movement of ions, stratum corneum, improved systemic bioavailability

## Abstract

Recently there has been an increased interest in using iontophoretic technique for the transdermal delivery of medications, both ionic and nonionic. This article is an overview of the history of iontophoresis and factors affecting iontophoretic drug transfer for the systemic effects and laws for development of Transdermal delivery system are discussed.

The benefits of using transdermal drug delivery include improved systemic bioavailability resulting from bypassing the first metabolism. Variables due to oral administration, such as pH, the presence of food or enzymes and transit times can all be eliminated. In the development of new transdermal drug delivery devices the aim is to obtain controlled, predictable and reproducible release of drugs into the blood stream of the patient. The transdermal device acts as a drug reservoir and controls the rate of drug transfer. When the transdermal drug flux is controlled by the device instead of the skin, delivery of the drug is more reproducible leading to smaller inter and intrasubject variations, since the drug release from the device can be controlled accurately than the permeability of the skin^1^.

The method of iontophoresis was described by Pivati in 1747. Galvani and Vota two well known scientists working in the 18^th^ century combined the knowledge that the electricity can move different metal ions and the movement of the ions produce electricity. The method of administering pharmacological agents by iontophoresis became popular at the beginning of 20^th^ century due to the work of Leduc (1900) who introduced the term iontotherapy and formulated the laws for this process.

Intophoresis is defined as the introduction, by means of a direct electrical current of ions of soluble salts into the tissue of the body for therapeutic purposes. It is a technique used to enhance the absorption of drugs across biological tissues such as the skin.

## AN OVERVIEW OF IONTOPHORESIS

Iontophoresis is the method where the movements of ions across a membrane enhanced using an externally applied potential difference. When the membrane under consideration is skin, the method is called transdermal iontophoresis. The principle barrier to the transport of the molecules into an across the skin is stratum corneum (SC), this is the uppermost layer of the epidermis with a thickness of between 10-100 μm. The SC consists of several layers of corneocytes (a nucleate keratin filled cells) inlaid in a lipid matrix, a continuous medium through the SC, arranged mainly in bilayers^2^,^3^. The intercellular lipids consist of approximately equal quantities of ceramides, cholesterols and free fatty acids^4^.

Percutaneous absorption may take place simultaneously by any combination of the three main pathways^4^–^7^ that include; the intercellular (paracellular) pathway between the conneocytes along the lamellar lipids, the intracellular (transcellular) pathway through the cells or the appendageal (shunt) pathway via hair follicles, sweat ducts and secretary glands.

Ions prefer the routes of the least electrical resistance; in the SC this is believed to be via the pores. Some investigations indicate that these pores are sweat glands^8^,^9^, others that transport occurs through both hair follicle and sweat glands^10^–^12^.

The physicochemical properties of the molecules have an effect on the contribution of the follicular and non follicular routes of penetration. Hydrophilic molecules tend to localize in the hair follicles, whereas lipophilic molecules are mostly distributed in the lipid intercellular regions of the SC and the lipid membranes of the epidermal keratinocytes^13^. Since passive transdermal permeation of the majority of the drugs needs enhancement to achieve clinically relevant plasma concentrations, both chemical and physical enhancement methods have been developed. Iontophoresis is one of the physical methods.

**Fig. 1:**
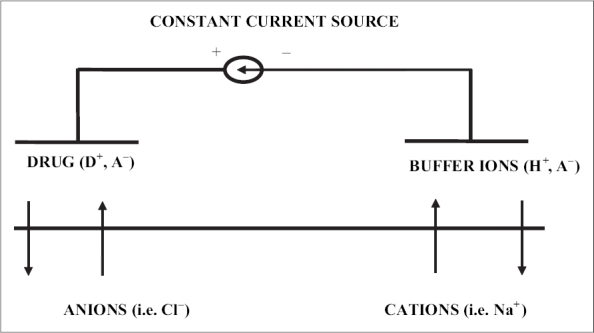
Transdermal iontophoretic system.

In iontophoresis, cationic or neutral therapeutic agents are placed under an anode or anionic therapeutic agents under a cathode. When a low voltage and low current density is applied, according to simple electrorepulsion, ions are repelled into and through the skin. Cationic drugs are driven into and through the skin by the anode (active electrode), which also extracts anion from the tissue underneath the skin into the anode. At the cathode (return electrode) anionic buffer ions are driven into the skin and cations from the tissues are extracted into the cathode. (fig. 1) It is also possible to include an additional charged drug in the return electrode to be delivered simultaneously or to use a mixture of drugs in the active electrode to enhance the desired effect or to increase skin permeation, depending on which drugs/molecules are used^14^–^16^.

More formally, transdermal iontophoresis should be called electrically assisted transdermal delivery. There are three major enhancing mechanisms for drug flux through the skin, of which iontophoresis (also known as electrorepulsion, or electromigration or the Nernst-Planck effect) is just one. The other mechanisms are electoroosmotic flow^17^–^21^ and current induced increase in skin permeation, also known as damage effect^22^. Electroosmotic flow is a flux or bulk fluid induced by a voltage difference across a charged membrane; it is always in the same direction as the flow of counter ions. Since human skin is negatively charged under physiological conditions, the counter ions are cations and the electroosmotic flow is thus from anode to cathode. Therefore, the cathodic delivery of anions is hindered and the anodic delivery of cations is assisted by electrosmosis.

The improved movement of neutral molecules under iontophoresis is based on electroosmosis. Ions are influenced by all of the above mechanisms so that electroosmosis has a positive contribution to the transport of cations and a negative contribution to the transport of anions under normal physiological conditions. The impact of electroosmosis on ion transfer increases with the size of the ion^23^. The contribution of electroosmosis can be so significant that the delivery of large anion from the anodic compartment can be more efficient than delivery from cathode, this is called wrong-way iontophoresis^19^.

The electrorepulsion effect gives the largest enhancement to the flux of small lipophillic cations^24^. When the concentration of the ionic drug is very high, so that the drug carries most of the current, electroosmotic flow has a very small effect on the drug flux^17^.

Transdermal iontophoresis has been used for both local and systemic drug delivery. Applications include local delivery of anaesthetics (e.g. lidocaine)^25^, steroids and retinoids to treat acne scarring^26^, for the relief of palmar and plantar hyperhidrosis^27^, and the administration of pilocarpine in the diagnosis of cystic fibrosis^28^–^30^. Other applications of transdermal iontophoresis include the administration of antiinflammatory drugs e.g. ketoprofen^31^, in to subcutaneous tissues and joints. Iontophoretic delivery of several systemic drugs is still under investigation. These include the analgesic, fentanyl^32^, a reversible cholinesterase inhibitor, tacrine III and several formulations of insulin^33^–^38^. The symmetrical nature of iontophoresis, where ions are driven both into and out of the body, has been utilized for extracting information from the body without the need for blood sampling.

## HISTORY OF TRANSDERMAL DELIVERY SYSTEM

The first proposal for the use of electric current in the drug delivery dates from the mid 18^th^ century. Serious progress was made in the 19^th^ century notably by Benjamin Ward Richardson (1828-1896). Hermann Munk (1839-1912), William James Morton (1846-1920), Stephen Leduc (1853-1939) and Fritz Frankenhauser (born 1868). Administration of metal ions as well as alkaloids was tried at that time. Until the early 20^th^ century, current medicated drug delivery was known as “cataphoresis”. Frankenhauser is said to have introduced the term “iontophoresis” before 1908. Recently researchers talk about “electrically-assisted transdermal drug delivery”. The technique was never widely adopted but always proved useful to some extent in solving particular drug delivery problems.

Twenty two years ago, the first transdermal drug delivery system was introduced in the US making a historic breakthrough, holding the promise that new compounds could be delivered in a safe, convenient way through this skin. And yet, during the last two decades, the commercial success of transdermal delivery has been slow to develop. But, as a spate of newer products and technologies move towards the market place, transdermal drug delivery seems to have arrived.

America's first commercially marketed transdermal patch used a passive mode of drug delivery that permitted the drug to diffuse through the avascular dermis to the deep dermis, allowing local action or penetration to the capillaries for a systemic effect. But these passive systems had limitations. This approach depended on the drug's properties to facilitate transport through the skin by using a simple concentration gradient as a driving force. Also, few drugs were available with the right physicochemical properties to make good candidates for transport through the skin. Even with these limitations, passive transdermal patches are experienced ever- increasing acceptance today.

While passive transdermal technology grows in popularity, all the available transdermal delivery systems use passive technology. Passive technology has always depended on the physicochemical properties of the drug candidate, large molecule drugs, such as, proteins and peptides, could not be considered. But, advances in the research have led to a better understanding of the physiology of the skin and more familiarity with the drug transport characteristics.

## FACTORS AFFECTING IONTOPHORESIS TRANSPORT

Many factors have been shown to affect the results of iontophoresis. These include the physicochemical properties of the compound (molecular size, charge, concentration), drug formulation (types of vehicle, buffer, pH, viscosity, presence of other ions), equipment used (available current range, constant *vs*. pulsed current, type of electrode), biological variations (skin site, regional blood flow, age, sex), skin temperature and duration of iontophoresis. The following factors have to be considered:

### Influence of pH:

The pH is of importance for the iontophoretic delivery of drugs. The optimum is a compound that exists predominantly in an ionized form. When the pH decreased, the concentration of hydrogen ion increases and a vascular reaction (vasodilatation) is initiated because of C-fiber activation, thus it is important to keep the pH as close as possible to and, at least when working with vasodilators, at pH 5.5 and below. There is an increasing risk for vascular reaction due to the high concentration of hydrogen ions rather than the compound used. Since hydronium ions are small they penetrate the skin more easily than larger drug ions. Laboratory findings vary on the effect of pH and drug behavior. According to the Henderson-Hasselbalch equation, pH is the determining factor governing the amount of drug present in the ionized state. For optimum IP, it is desired to have a relatively large proportion of the drug in the ionized state. However, this must be counterbalanced with delivery of a drug at a pH that is tolerable and safe for the patient^39^.

### Current strength:

There is a linear relation between the observed fluxes of a 1-cm^2^, the current is limited to 1 mA due to patient comfort considerations. This current should not be applied for more than 3 min because of local skin irritation and burns. With increasing current, the risk of non specific vascular reactions (vasodilatation) increases. At a current of 0.4-0.5 mA/cm^2,^ such a vascular reaction is initiated after a few seconds of iontophoresis with deionised or tap water. This latter effect is probably due to current density being high enough a small area to stimulate the sensory nerve endings, causing reactions such as the release of substance P from C-fiber terminals^40^,^41^.

### Current density:

Current density is the quantity of current delivered per unit surface area. The following criteria should be considered in selecting proper current densities for IP. The current should be sufficiently high to provide a desired drug delivery rate. It should not produce harmful effects to the skin. There should be a quantitative relationship between the applied current. The drug should be electrochemically stable.

### Ionic competition:

In a solution of sodium chloride, there is an equal quantity of negative (Cl^−^) and positive (Na^+^) ions. Migration of a sodium ion requires that an ion of the opposite charge is in close vicinity. The latter ion of opposite charge is referred as a counter-ion. An ion of equal charge but of different type is referred as a co-ion. When using iontophoresis, it is important to know that pH adjustment is performed by adding buffering agents. The use of buffering agents as co-ions, which are usually smaller and more mobile than the ion to be delivered results in a reduction of the number of drug ions to be delivered through the tissue barrier by the applied current. In our example, this means that when a positively charged drug is diluted in saline, the sodium ions will compete with the amount of drug ions to be delivered. Ideally, the use of a buffer system should be avoided in iontophoresis, but if this is not possible, alternative buffers, consisting of ions with low mobility or conductivity are preferred^42^.

### Drug concentration:

Depending on the drug used, the steady-state flux (ion movement) has been shown to increase with increasing concentration of the solute in the donor compartment, i.e. in the delivery electrode. Increased uptake by the skin during and after IP with an increase in drug concentration has been reported^43^,^44^. A limiting factor to be considered is the strength of the current used. At higher drug concentration, probably because of the saturation of the boundary layer relative to the donor bulk solution (Phillips *et al.* 1989).

### Molecular size:

It has been shown that the permeability coefficients in positively charged, negatively charged and uncharged solutes across human skin are a function of molecular size. When the molecular size increases, the permeability coefficient decreases. However, there are certain solutes with a relatively high molecular size (e.g. insulin, vasopressin and several growth hormones), which have also been to penetrate the skin barrier into the systemic circulation^45^.

### Connective or electro-osmotic transport:

When performing iontophoresis with a specific current, the flow of ions across the membrane induces a flow of solvent called electro-osmosis. Compared to the ion transport, the electro-osmotic contribution is small. The penetration of uncharged substances (e.g. bovine serum albumin) has been shown to be facilitated by the volume flow effect induced by an applied potential difference across the membrane. Iontophoresis has also been observed to enhance the penetration of a number of dipolar ions (zwitter ionic substances like phenylalanine). Most of these substances have been shown to be delivered in significantly higher amounts by anodic delivery than by cathodic delivery. In general, iontophoresis is more effective for charged compounds, especially monovalent ions.

### Current-continuous *vs*. pulsed mode:

Application of a continuous current over a long period of time can modulate iontophoresis delivery. Continuous DC current may result in skin polarization, which can reduce the efficiency of iontophoretic delivery in proportion to the length of current application. This polarization can be over come by using pulsed DC, a direct current that is delivered periodically. During the ‘off time” the skin becomes depolarization using pulsed DC can, however, decrease the efficiency of pulsed transport if the frequency is too high. Enhanced iontophoretic transport has been reported for peptides and proteins by using pulsed Dc compared to convenient DC. Most of the drug ions used for diagnostic purposes in combination with iontophoresis and LDPM are small in size. As a result, the time needed for an effect is relatively short (5-20 s) compared to when iontophoresis is used for therapeutic purposes (20-40 min).

### Physical factors:

Iontophoresis reduces intra and inter-subject variability in the delivery rate. This is an inherent disadvantage with the passive absorption technique. Experiments *in vivo* iontophoretic give support for clinical findings that there are small differences in the flux rate following transdermal iontophoresis between males and females, as well as between hairy and hairless skin. The status of the vascular bed is also important; for instance, a pre-constricted vascular bed decreases the flux through the skin while a dilated vascular bed increases the yield of the drug through the skin.

### Drug salt form:

It has been reported^46^ that different salt forms have different specific conductivities and that conductivity experiments *in vitro* will provide information concerning the general suitability of a drug for IP. The salt form of drugs must be considered along with the pH of the solution for determining the amount of drug in the ionized state.

### Patient anatomical factors:

Patient anatomical factors that influence the depth of penetration that is variable from patient to patient include skin thickness at the site of the application, presence of subcutaneous adipose tissue and the size of other structures, including skeletal muscle. Additionally, the presence and severity of inflammation can influence drug penetration due to the increased temperature (which may increase and may serve to transport the drug throughout the body^47^.

### Type of matrix containing the drug, gel *vs*. solutions:

The migration of the drug under the influence of the electrical current will be different as the matrices are different. This can be related to differences in viscosities, material electrical charge and porosities.

### Stability of the drug during the IP process:

The drug undergoing IP must be stable in the solution environment up to the time of Ip and also during the iontophoresis process. Oxidation or reduction of a drug not only decreases the total drug available but the degradation compounds, if they posses the same charge as the drug ion, will complete with the drug ion and reduce the overall trans membrane rate of the drug.

### Laws for the development of transdermal drug system^48^:

Transdermal drug delivery systems follow the general law of developing and evolving according to an “S-Curve” profile (the plot of a major index of the system performance versus time). All transdermal systems consist of four essential parts, an energy source, a sub-system that transmits system energy to those locations where it is required for performance, the part (or parts) that actually accomplish the main function of the system, and, a control system that monitors and controls system functioning. These four essential parts need to be complete in order for the system to function at a high level. A most important aspect of the further development of transdermal drug delivery systems will be breakthroughs in how effectively energy is transmitted throughout the system. There are six measures of ideality of transdermal drug delivery systems. These can be used as predictive gauges to indicate where a particular transdermal delivery system is on its S-curve and the next developmental design step. Transdermal drug delivery systems of the future will minimize human involvement, and will even include additional features and functions previously requiring human actions. There will always be a specific sub-system that represents the largest opportunity for system development. This opportunity can be identified by through the use of technical innovation algorithm (TIA). Transdermal drug delivery systems developed by traditional approaches will become far more complex in their construction (this is not true for TIA-developed systems, which stress system and elegance). An important developmental direction that transdermal drug delivery systems will take is the path of increasing flexibility, controllability, directability and adjustability. Major breakthroughs in transdermal drug system designs will occur because of the introduction of new, modified energy sources (detailed information about this law may not be provided because of its highly proprietary nature). There is a law that defines the relationship between transdermal drug delivery systems and other existing and new systems. Next generation transdermal drug delivery systems will show improved degrees of coordination among certain system parts, and intentional dis-coordination among other system parts. The purpose of this coordination or dis-coordination by design is to achieve significant breakthroughs in overall system performance.

## CONCLUSION

Traditional transdermal patches have been available for more than 20 years, and they have a proven history of success. With these new technologies, the number and complexity of transdermal drug delivery systems will increase in the near future. Pharmacists who become familiar with these technologies will be better able to address patient questions and concerns.

## References

[1] Guy RH, Hadgraft J (1992). Rate control in transdermal drug delivery. Int J Pharm.

[2] Bouwstra JA, De Vries MA, Gooris GS, Bras W, Brussee J, Ponec M (1991). Thermodynamic and structural aspects of the skin barrier. J Control Release.

[3] Elias PM (1991). Epidermal barrier function: Intercellular lamellar lipid structures, origin, composition and metabolism. J Control Release.

[4] Schnetz E, Fartasch M (1991). Microdialysis for the evaluation of penetration through the human skin barrier: A promising tool for future research. Eur J Pharm Sci.

[5] Singh J, Bahtia KS (1996). Topical iontophoretic drug delivery, Pathways, principles, factors and skin irritation. Med Res Rev.

[6] Cullander C (1992). What are the pathways of iontophoretic current flow through mammalian skin?. Adv Drug Del Rev.

[7] Riviere JE, Heit MC (1997). Electrically-assisted transdermal drug delivery. Pharm Res.

[8] Abramson HA, Gorin HH (1940). Skin reactions, IX the electrophoretic demonstration of the patient pores of the living human skin, its relation to the charge of the skin. J Phys Chem.

[9] Grimnes S (1984). Pathways of ionic flow through human skin *in vivo*. Acta Derm Venereol.

[10] Burnette RR, Marrero D (1986). Comparision between the iontophoretic and passive transport of thyrotropin releasing hormone across existed nude mouse skin. J Pharm Sci.

[11] Del Terzo S, Bhel CR, Nash RA (1989). Iontophoretic transport of a homologues series of ionized and nonionized model compounds; Influence of Hydrophobicity and mechanistric interpretation. Pharm Res.

[12] Burettee RR, Ongipipattankul B (1988). Characterization of the pore transport properties and tissue alteration of excised human skin during iontophoresis. J Pharm Sci.

[13] Turner NG, Guy R (1997). Iontophoretic transport pathways, dependence on penetrant physiochemical properties. J Pharm Sci.

[14] Deagle WR (2003). Iontophoresis dis pain blocker, U.S. patient No. US2003100884, acc.

[15] Riviere JE, Sage B, Williams PL (1991). Effects of vasoactive drugs on transdermal lidocaine iontophoresis. J Pharm Sci.

[16] Riviere JE, Monteiro NA, Inman AO (1992). Determination of lidocaine concerntrations in skin after transdermal iontophoresis: Effects of vasoactive drugs. Pharm Res.

[17] Pikal MJ, Shah S (1990). Transport mechanisms in iontophoresis II. Electroosmotic flow and transference number measurement for hairless mouse skin. Pharm Res.

[18] Pikal MJ (1990). Transport mechanisms in iontophoresis, I. A theoretical model for the effect of electroosmotic flow on flux enhancement in transdermal iontophoresis. Pharm Res.

[19] Pikal MJ (2001). The role of electroosmosic flow in transdermal iontophoresis. Adv Drug Del Rev.

[20] Uitto OD, White S (2003). Electrosmotic pore transport in human skin. Pharm Res.

[21] Pikal MJ (1992). The role of electroosmosic flow in transdermal iontophoresis. Adv Drug Del Rev.

[22] Inada H, Ghanem AH, Higuchi WI (1994). Studies on the effects of applied voltage and duration on human epidermal membrane alteration/recovery and the resultant effects upon iontophoresis. Pharm Res.

[23] Panchagula R, Pillai O, Nair VB, Ramanrao P (2000). Transdermal iontophoresis revisited. Curr Opin Chem Boil.

[24] Marro D, Kalia YN, Delgado-Charro MB, Guy R (2001). Contributions of electromigration and electroosmosis to iontophoretic drug delivery. Pharm Res.

[25] Maloney JM (1992). Local anesthesia obtained via iontophoresis as an aid to shave biopsy. Arch Dermatol.

[26] Schmidt JM, Binder M, Maicheiner W, Bielglmayer C (1995). New treatment of atrophic acne scars by iontophoresis with estriol and tretinoin. Int J Dermatol.

[27] Sloan JB, Soltani K (1986). Iontophoresis in dermatology. J Am Acard Dermatol.

[28] Gibson LE, Cooke RE (1959). A test for concentration of electrolytes in sweat in cystic fibrosis of the pancreas utilizing pilocarpine by iontophoresis. Pediatrics.

[29] Gibson LE (1967). Iontophorestic sweat test for cyctic fibrosis, technical details. Pediatrics.

[30] Webster HL, Barlow WK (1981). New approach to cystic fibrosis diagnosis by use of an improved sweat-induction/collection system and osmometry. Clin Chem.

[31] Panus PC, Campbell J, Kulkarni SB, Herrick R, Ravis WR, Banga AK (1997). Transdermal iontophoretic delivery of ketoprofen through human cadaver skin and in humans. J Control Release.

[32] Ashburn MA, Streisand J, Zhang J, Love G, Rowin M, Niu S (1995). The iontophoresis of fentanyl citrate in humans. Anesthesiology.

[33] Chein YW, Siddique O, Shi WM, Lelawongs P, Liu JC (1989). Direct current intophoretictransdernal delivery of peptide and protein drugs. J Pharm Sci.

[34] Stepen RL, Petelenz TJ, Jacobsen SC (1984). Potential novel methods for insulin administration: I Iontophoresis. Biomed Biochem Acta.

[35] Pillai O, Borkute SD, Sivaprasad N, Panchagnula R (2003). Transdermal iontophoresis of insulin II: Physiochemical considerations. Int J Pharm.

[36] Pillai O, Nair V, Panchagnula R (2004). Transdermal iontophoresis of insulin IV: Influence of chemical enhancers. Int J Pharm.

[37] Pillai O, Panchagnula R (2003). Transdermal iontophoresis of insulin V: Efeect of terpenes. J Controlled Release.

[38] Rastogi SK, Singh J (2002). Transepidermal transport enhancement of insulin by lipid extraction and iontophoresis. Pharm Res.

[39] Fang JY, Hung CF, Wong WW (2006). Skin Pharmacol Physiol.

[40] Abramowitz D, Neoussikine B (1946). Treatment by Ion Transfer.

[41] Schriber WJ (1975). A manual of electrotherapy.

[42] Bumette RR, Ongpipattanakul B (1988). Charecterization of the pore transport properties and tissue alteration of excised human skin during iontophoresis. J Pharm Sci.

[43] Siddiqui O, Roberts MS, Polock AZ (1985). The effect of iontophoresis and vehicle pH on the in-vitro permeation of lignocaine through human stratum corneum. J Pharm Pharmacol.

[44] Abramson HA, Alley A (1937). Mechanisms of histamine iontophoresis from aqueous media. Arch Phys Ther.

[45] Chien YW, Siddiqui O, Shi W, Lelawongs P, Liu JC (1989). Direct current iontophoretic Transdermal delivery of peptides and protein drugs. J Pharm Sci.

[46] Gangarosa LP, Park NH, Fong BC, Scott DF, Hill JM (1978). Conductivity of drugs used for iontophoresis. J Pharm Sci.

[47] Singh P, Maibach HI (1994). Iontophoresis in drug delivery; Basic principles and applications. Crit Rev Ther Drug Car Sys.

[48] http://www.triz.journal.com/archives/1997/12/f/.

